# A Rare Case of Infective Endocarditis Caused by Gemella haemolysans

**DOI:** 10.7759/cureus.6234

**Published:** 2019-11-26

**Authors:** Tanushree Agrawal, Malcolm Irani, Stephanie Fuentes Rojas, Omar Jeroudi, Ejaz Janjua

**Affiliations:** 1 Internal Medicine, Houston Methodist Hospital, Houston, USA; 2 Cardiology, Debakey Heart and Vascular Center, Houston Methodist Hospital, Houston, USA

**Keywords:** infective endocarditis, infective endocarditis, bicuspid aortic valve, surgical aortic valve replacement, gemella haemolysans

## Abstract

Gemella haemolysans is a gram-positive coccoid, facultative anaerobe of the mucous membranes. In rare cases, it has been identified as an opportunistic pathogen in the development of endocarditis. Here, we describe a case of infective endocarditis in a patient with a bicuspid aortic valve. A 38-year-old man presented with the complaint of exertional dyspnea of one month duration. He was found to have leucocytosis and his blood cultures grew Gemella haemolysans. Trans-esophageal echocardiography showed a bicuspid aortic valve with 1.5 x 1.5 cm vegetative mass, severe aortic regurgitation, and an aortic root abscess. The patient was started on intravenous ampicillin and gentamycin. He then underwent mechanical aortic valve replacement and bovine reconstruction of the left ventricular outflow tract. Our case highlights the importance of considering atypical pathogens as causative agents of infective endocarditis.

## Introduction

Infective endocarditis is a rare disease, associated with a high burden of morbidity and mortality. The incidence of infective endocarditis in the United States has been reported to be around 15 per 100,000 population [1]. The most commonly reported micro-organisms causing infective endocarditis include Staphylococcus aureus, coagulase negative Staphylocci, Viridans group Streptococci, Streptococcus bovis, Enterococci [2]. The diagnosis of infective endocarditis is often challenging, and even more so, when it involves atypical organisms. We present here a case of infective endocarditis caused by Gemella haemolysans in a patient with bicuspid aortic valve.

## Case presentation

**Figure 1:**
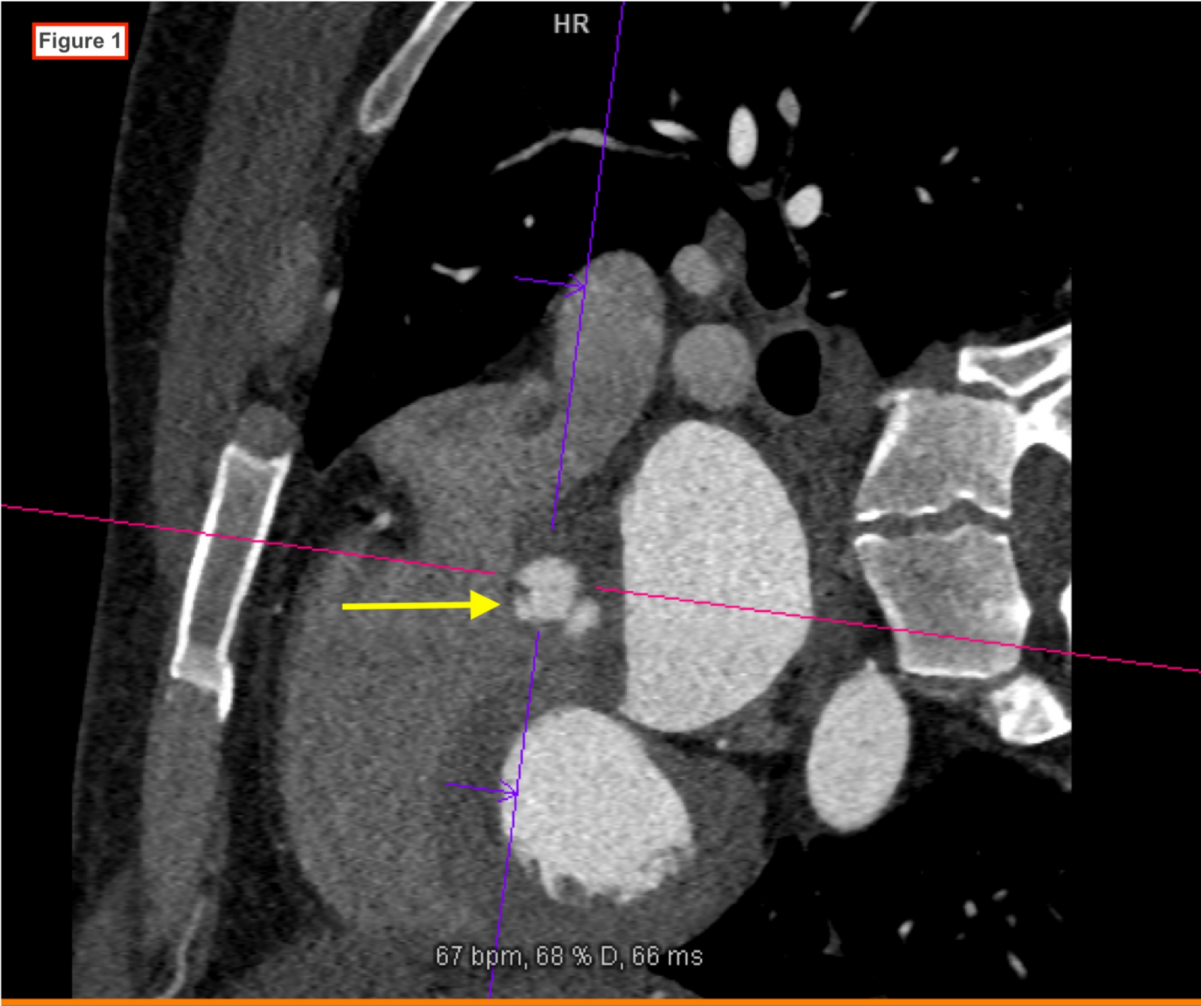
Cardiac CT image showing a 3 x 5 mm vegetation on the non-coronary leaflet of the aortic valve.

**Figure 2:**
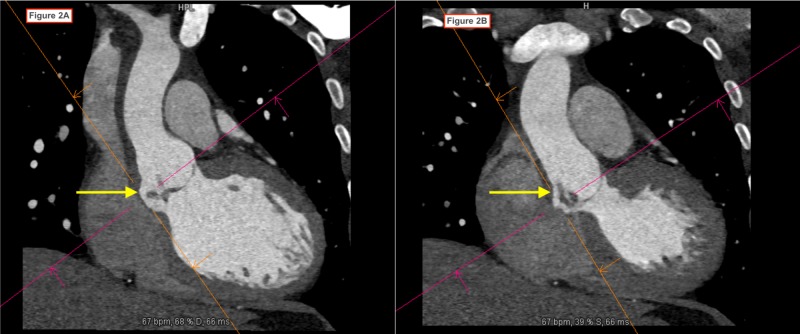
Cardiac CT scan showing aortic root pseudo-aneurysm involving the non-coronary sinus of Valsalva with a fistula from the aortic root to the left ventricle.

**Figure 3:**
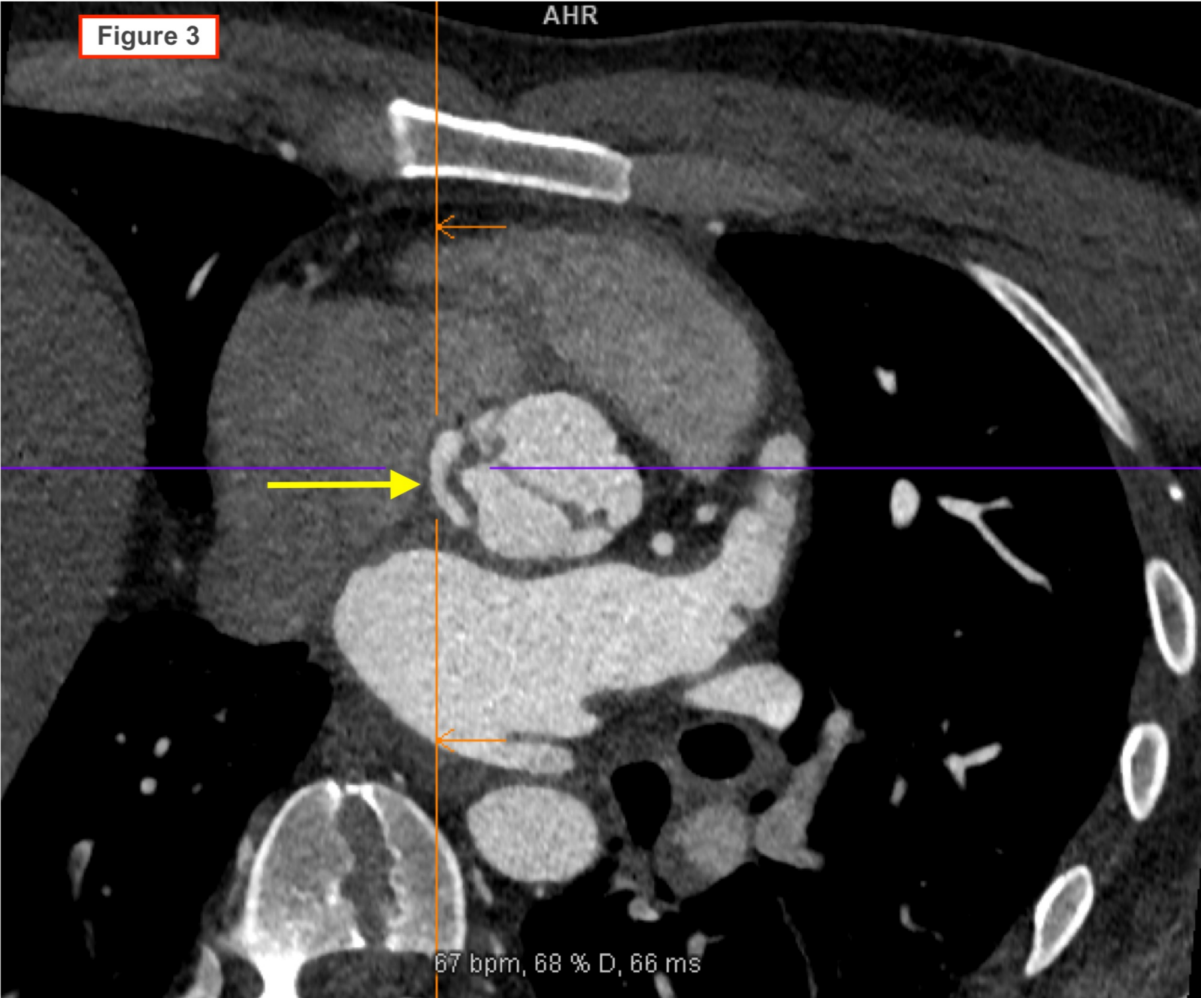
Cardiac CT image showing a fistula from the base of the left ventricle into the left atrium.

A 38-year-old man with a history of chronic mastoiditis was admitted to the hospital with the chief complaint of exertional dyspnea of one month duration. He denied any history of chest pain, palpitations or lower extremity swelling. He had no recent history of fever and he denied any history of illicit drug use. He denied ear pain, tooth pain, or recent dental work. On admission, he was afebrile and vital signs were within normal limits. His cardiovascular examination did not reveal jugular venous distention (JVD) or pitting edema and a murmur was not appreciated. His lungs were clear to auscultation bilaterally. Laboratory studies were notable for the presence of white blood cell count 20,170/mm^3^ (91% neutrophils). The initial electrocardiogram (EKG) showed sinus tachycardia (HR 102 bpm) with a first-degree AV block (PR interval 244 ms). During his workup for leukocytosis, peripheral blood cultures grew Gemella haemolysans in two out of two cultures. A transthoracic echocardiogram (TTE) showed left ventricular ejection fraction 60-64%, mild left ventricular concentric remodeling, an enlarged left atrium, poorly visualized aortic valve root with findings suspicious for aortic regurgitation. Due to poor visualization of the aortic valve, a transesophageal echocardiogram (TOE) was performed the next day. It showed a bicuspid aortic valve with a 1.5 x 1.5 cm vegetative mass attached to the non-coronary cusp with resultant severe aortic regurgitation, an aortic root abscess, and two vegetations along the ventricular aspect of the mitral valve. These findings were confirmed on cardiac CT scan (Figures 1-3). A diagnosis of infective endocarditis (IE) was made and the patient was started on IV ampicillin (2 grams every six hours) and gentamicin (240 milligrams daily). After initiation of antibiotic treatment, repeat blood cultures drawn on day 3 were negative and on day 6 he underwent surgical treatment of the aortic valve endocarditis. Intraoperatively, no abscess was seen. However, there were vegetations on both cusps of the aortic valve, moth-eaten appearance of the intervalvular fibrosa between the aortic and mitral valves, and a fistulous connection from the left ventricle to the right ventricle. Mechanical aortic valve replacement and bovine reconstruction of the left ventricular outflow tract was performed. He was subsequently started on anticoagulation with warfarin. He was discharged after sterilization of blood cultures with three weeks of intravenous antibiotics from the date of the first negative culture. Further workup for the source of infection included a panorex X-ray that showed a periapical abscess at the root of the left mandibular wisdom tooth. He underwent removal of six teeth 15 days after the valve replacement surgery.

## Discussion

Gemella haemolysans is a gram-positive coccoid microbe. It decolorizes easily in the Gram stain and sometimes appears Gram-negative or Gram-variable [3]. It was initially named “Neisseria haemolysans” in 1938, but was later reclassified to genus Gemella based on enzymatic studies that showed it to be catalase negative and oxidase negative [4]. It is a facultative anaerobe and lives as a commensal in the upper respiratory, gastrointestinal and genitourinary tracts of humans [5]. Risk factors associated with invasive infections by Gemella haemolysans include immunosuppression, poor dentition, valvular heart disease, and prosthetic joints. Literature review reveals that Gemella haemolysans endocarditis typically involves native as well as prosthetic aortic and mitral valves [6]. Buu-Hoi et al. isolated Gemella haemolysans from patients with subacute endocarditis and determined the minimal inhibitory concentrations of 21 antimicrobial agents. Penicillin was found to exhibit synergism with aminoglycosides and therefore this combination is recommended for the treatment of Gemella haemolysans infective endocarditis [7].

In our patient, the presence of first-degree atrioventricular block in the setting of bacteremia raised concern for infective endocarditis. In a retrospective review of 142 patients with infective endocarditis, Wang et al. reported that 4% of the patients had complete heart block and 10% had incomplete heart block [8]. They found that these patients were more likely to have aortic valve involvement and peri-valvular extension of the inflammatory process. Therefore, the finding of conduction abnormalities in the setting of infective endocarditis should alert one to the possibility of peri-valvular extension of the infection.

Interestingly, the fistulous connection between the left ventricle and right ventricle noted during surgery, was not seen on imaging. A negative early trans-esophageal echocardiogram does not exclude the potential for development of a peri-valvular fistula or pseudoaneurysm, which develops over time. Many studies have compared the sensitivity and specificity of TTE and TOE in the diagnosis of intra-cardiac vegetations. In the majority of these studies the sensitivity of trans-thoracic echocardiography ranges between 40-63% and that of trans-esophageal echocardiography between 90-100% [9]. It is of vital importance to detect intra-cardiac abscesses early because they herald an ominous prognosis. Intra-cardiac abscesses represent areas that are not penetrated by antibiotics, therefore warranting early surgical treatment.

A retrospective review published in 2009 found the presence of bicuspid aortic valve to be the only significant independent predictor of peri-annular complications in patients with infective endocarditis. Patients with bicuspid aortic valves not only face a higher risk of infective endocarditis, but they also have a higher likelihood of developing peri-annular complications such as abscess, pseudoaneurysm, aortocavitary fistulae [10]. A retrospective analysis of 50 patients with bicuspid aortic valve infective endocarditis reported a high incidence of serious complications - 30% of the patients had peri-annular abscess formation, 72% developed heart failure and 82% of the patients required surgery [11].

Indications for valvular heart surgery in patients with infective endocarditis include heart failure, abscess formation, heart block, persistent bacteremia lasting more than seven days after appropriate antibiotic therapy, recurrent systemic emboli and persistent or enlarging vegetations despite appropriate antibiotic therapy [12]. Our patient had an aortic root abscess noted on echocardiography and this warranted surgical treatment.

## Conclusions

We learned from this case that it is important to keep a high index of suspicion for infective endocarditis in the setting of bacteremia with atypical micro-organisms. Gemella haemolysans, although a rare pathogen, led to significant structural damage in the case of our patient. It is also very important to perform trans-esophageal echocardiography when the trans-thoracic echocardiography findings are equivocal. Early diagnosis, appropriate antibiotics and prompt surgical management led to a favourable outcome in our patient. To the best of our knowledge, this is the first case report of infective endocarditis caused by Gemella haemolysans in a patient with bicuspid aortic valve.
